# Colorado stride (COSTRIDE): testing genetic and physiological moderators of response to an intervention to increase physical activity

**DOI:** 10.1186/1479-5868-10-139

**Published:** 2013-12-21

**Authors:** Angela D Bryan, Renee E Magnan, Ann E Caldwell Hooper, Joseph T Ciccolo, Bess Marcus, Kent E Hutchison

**Affiliations:** 1 University of Colorado Boulder, Boulder, CO 80309, USA; 2 Washington State University Vancouver, Vancouver, WA 98686, USA; 3 University of New Mexico, Albuquerque, NM 87131, USA; 4 Columbia University, New York, NY 10027, USA; 5 University of California San Diego, La Jolla, CA 92093, USA

**Keywords:** Exercise, Physical activity, Intervention, Genetics, Transdisciplinary

## Abstract

**Background:**

The purpose of this research was to replicate a successful intervention to increase physical activity in a different region of the country, and explore genetic and physiological moderators of intervention efficacy drawn from a transdisciplinary theoretical framework.

**Method:**

A randomized controlled trial comparing a print-based physical activity intervention (COSTRIDE) to a print-based health and wellness contact control (HW) intervention was conducted. Sedentary participants (*n* = 219) completed assessments at baseline and follow-up assessments at 3, 6, 9, and 12 months following the initiation of the intervention.

**Results:**

Participants in both conditions significantly increased exercise behavior in the first six months, and then leveled off or decreased physical activity in the second six months of the study. Those in the COSTRIDE intervention increased significantly more than those in the HW intervention, and were better able to maintain their exercise behavior. Genetic factors (*BDNF*, rs6265; *FTO*, rs8044769), but not selected physiological (body temperature, blood lactate, systolic blood pressure, plasma norepinephrine, and heart rate) or subjective (perceived pain, affect) responses to physical activity, moderated response to the intervention.

**Conclusions:**

There are underlying genetic factors that influence response to behavioral intervention, and a better understanding of these factors has the potential to influence the development, targeting and tailoring of behavioral interventions to increase physical activity.

**Trial registration:**

Clinicaltrials.gov registration: NCT01091857.

## Background

Regular physical activity has been implicated in the prevention of a number of cancers including those of the colon, breast, endometrium, and prostate [1]. In addition, physical inactivity accounts for one-third of all heart disease and Type II diabetes mortality [2]. Despite the benefits of physical activity, 75% to 95% of the U.S. population do not get the recommended amount as defined by 30 minutes of regular physical activity 5 or more days per week [3], and 36% are considered inactive [4]. The development of effective, theory-based physical activity promotion interventions is of paramount importance in order to decrease disease morbidity and mortality. Though interventions to increase physical activity show some success, the changes in behavior are small and difficult to maintain [5]. One potential reason for these modest effects is that there is likely not a “one size fits all” intervention to increase physical activity. A number of authors have highlighted the crucial importance of understanding individual differences that may moderate the effectiveness of physical activity interventions [5] and perhaps allow for targeted interventions to those individuals for whom they are most likely to work [6]. Recent commentary suggests that one approach to discovering potentially important moderators of intervention effectiveness is through a better understanding of the underlying genetic, biological, and cognitive determinants of physical activity [7].

**Figure 1:**
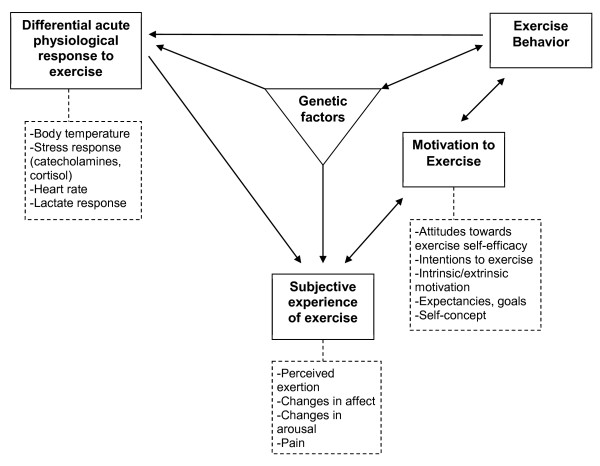
Transdisciplinary framework for exercise behavior.

In order to characterize the potential moderators of the effectiveness of physical activity interventions, we have proposed a transdisciplinary framework outlining mechanisms through which genetic and physiological factors might influence the subjective experience of exercise and, consequently, the motivation to engage in physical activity (see Figure 1) [8]. We utilized this theoretical conceptualization of the psychological, biological, and genetic determinants of exercise behavior to select a set of moderators we believed might moderate the effectiveness of a well-tested intervention to increase physical activity. The goal of the current study was twofold. The first aim was to replicate a previously successful intervention to promote physical activity among sedentary adults [9,10] in a different region of the country. The second aim was to explore potential moderators of intervention efficacy drawn from the theoretical framework.

### Current state of the art in physical activity intervention

In an effort to identify an efficacious physical activity intervention with the potential for wide scale dissemination, a wealth of non face-to-face interventions have been explored [11]. Non face-to-face interventions are a low-cost alternative to in-person counseling, are ideal for reaching a large number of individuals, and can be effective when individually tailored and grounded in theory [12]. A recent systematic review of tailored, print-based randomized controlled trials found positive results in seven out of 12 identified studies, with increases in physical activity ranging from one month to 24 months post-baseline [12]. One of the studies identified in that review was STRIDE [9], the 12-month print-based, individually tailored and theoretically grounded physical activity promotion program used in the current trial. STRIDE was tailored using theoretical constructs associated with the transtheoretical model (TTM) [13] and social cognitive theory (SCT; i.e. self-efficacy) [14] using a computerized expert system. In a randomized trial with sedentary adults comparing a Print-based to a Telephone-based STRIDE intervention, both conditions had significant increases in minutes/week of physical activity completed at 6 months relative to Control. However, physical activity continued to increase in Print between 6 and 12 months and was significantly greater than both Control and Phone at study end. Given these findings, STRIDE was chosen as the ideal physical activity intervention to use in this transdisciplinary study.

### Moderators of physical activity intervention effectiveness

Even when successful, physical activity interventions work better for some than for others [5], and we posited that this should also be true for STRIDE. A number of investigators have attempted to identify the moderators of response to intervention efficacy, with varying success. For example, Luszczynska and colleagues [15] demonstrated that perceived self-efficacy for health behavior change moderated the effectiveness of a planning intervention. Multiple studies have examined demographic variables that might moderate intervention efficacy. In a meta-analytic review of environmental interventions to change diet and exercise, Kremers and colleagues [16] noted that of 41 studies only seven explored moderators, most commonly gender, race, and age. There was evidence for interventions working somewhat better for women and ethnic minority adults. Burke and colleagues [17] examined a long list of moderating variables of the effectiveness of a lifestyle intervention among hypertensive individuals, and the only significant moderator of time spent in physical activity was gender. Women responded better than men. Yet in a meta-analysis, Conn and colleagues [18] found that participant characteristics (e.g., age, gender) were unrelated to the effect sizes of the interventions reviewed. To date, the state of the literature on potential moderators of the effectiveness of interventions is inconsistent, and there is often no strong rationale for the inclusion of the classes of moderators explored in these studies. Given the vastness of the *potential* moderating mechanisms underlying the effectiveness of these interventions we suggest that perhaps a more effective strategy is to begin with a mechanistic model of the underlying genetic, physiological, and affective determinants of the initiation and maintenance of exercise behavior.

### A transdisciplinary approach to the selection of moderators

We have proposed one possible way to organize these mechanistic variables in a transdisciplinary framework see (Figure 1) [19,20]. We culled different aspects of the experience of exercise across physiological and psychological constructs, and then posited a framework that attempts to link these physiological and psychological responses to their biological and genetic underpinnings. The framework suggests that genetic factors influence physiological responses to exercise. For example, we showed that two single nucleotide polymorphisms (SNPs) in the CAMP responsive element binding protein 1 gene (*CREB1* rs2253206 and rs2360969) were related to change in temperature during exercise, the SLIT2 SNP rs1379659 and the FAM5C SNP rs1935881 were associated with norepinephrine change during exercise, and a SNP in the μ–opioid receptor gene (*OPRM1*, rs1799971) was related to changes in norepinephrine and lactate [21]. The framework further suggests that genetic factors might be associated with subjective experiences of exercise. We have shown that a brain-derived neurotrophic factor (*BDNF*) SNP [19] and the OPRM1 SNP are associated with affective response to exercise [21]. Physiological responses are assumed to influence subjective experiences, and we have shown this to be the case [20] and those subjective experiences may influence the motivation to exercise. For example, we demonstrated that perceived pain and negative affect during exercise were associated with lower motivation to be physically active [20] while positive affect is associated with higher motivation to be active [22-24]. Motivation, in turn, is expected to influence behavior; a link that is supported by numerous meta-analyses and reviews e.g., [25,26]. Finally, the framework comes full circle to acknowledge that as exercise behavior increases it influences both physiological responses to exercise through training e.g., [27], and gene expression [28]. The framework is not a static and unidirectional representation of behavior at one point in time and therefore proposes two bi-directional paths. The framework proposes that not only will positive affective responses to exercise lead to increased motivation to exercise [8,22], but also that increased motivation influences affective responses to exercise [29]. Further, motivation may lead to more sustained exercise over time, but successfully engaging in exercise also likely increases motivation to continue [30].

All of the individual linkages and pathways proposed in the model have been supported empirically, and the implication of the framework, broadly speaking, is that the capacity to physiologically and psychologically adapt to and cope with physical activity may moderate the extent to which an individual increases their physical activity in response to an intervention. For example, an individual with a better catecholamine response (e.g., norepinephrine) to exercise may perceive exercise as more positive, thus experiencing differential immediate benefits of exercise which could translate into greater motivation to exercise and higher levels of exercise behavior.

Based on the extant literature and our prior analysis of the relationships among the variables identified in the framework [20], the moderators from the physiological responses to exercise domain included change during a bout of moderate intensity exercise in body temperature, blood lactate, systolic blood pressure, heart rate, and plasma norepinephrine. The moderators selected from the subjective experience of exercise domain were perceived pain and affect. Genetics factors were selected based on prior testing of the relationships between specific SNPs that were associated with physiological and subjective responses to moderate-intensity physical activity with individuals in separate analyses from this same sample [21]. These included three SNPs of the fat mass and obesity associated protein gene (*FTO*; rs9941349, rs8044769, and rs3751812), two SNPs of the*CREB1* gene; rs2253206 and 2360969), a SNP of the *OPRM1* gene (rs1799971), a SNP of the *FAM5C* gene (rs1935881), and a SNP of the *SLIT2* gene (rs1379659). See Karoly et al. [21] for an in-depth rationale of the selection of these SNPs. We also included one additional SNP from the brain-derived neurotrophic factor gene (*BDNF*; rs6265) based on our prior work [19]. All SNPs were in Hardy-Weinberg equilibrium (rs9941349, *p* = .140; rs8044769, *p* = .159; rs3751812, *p* = .119; rs2253206, *p* = .400; 2360969, *p* = .706; rs1799971, *p* = .529; rs1935881, *p* = .346; rs1379659, *p* = .068; and rs6265, *p* = .015). Minor allele frequencies for all SNPs except BDNF appear in Karoly et al. [21]. For BDNF: 62.6% were G/G, 26% were A/G, and 7.3% were A/A.

## Method

COSTRIDE was a 12-month randomized controlled trial (RCT) in which participants were randomly assigned to the print-based STRIDE exercise intervention (COSTRIDE) or a health-and-wellness contact control condition (HW). All participants completed three baseline sessions (orientation, fitness assessment, and submaximal exercise session), and follow-up assessments at 3, 6, 9, and 12 months following randomization. Extensive detail regarding the rationale for selection of moderators, recruitment, measures, intervention procedures, and baseline relationships in the data including among constructs in the transdisciplinary framework are available in Magnan et al. [20]. The goal of the analyses presented here is solely to assess intervention effects on behavior and potential moderators of those effects. This research was conducted at the University of Colorado at Boulder (CU) and was approved by all relevant review boards.

### Participants

**Figure 2:**
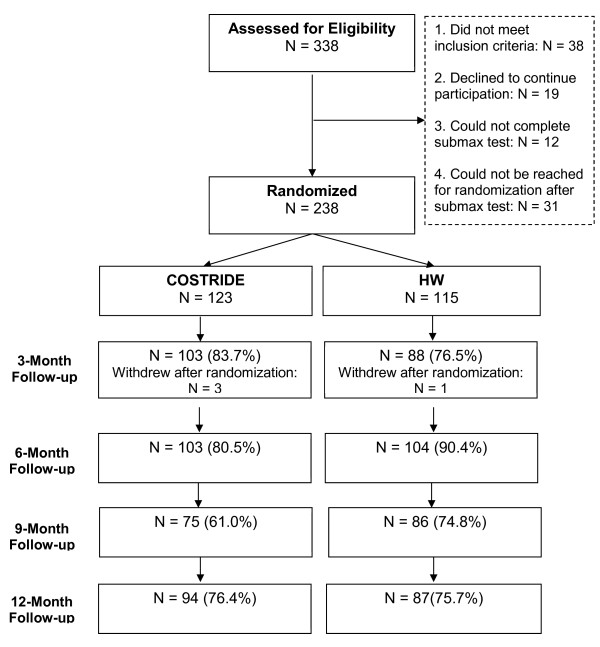
Consort chart for participant progression through the study.

Participants were men and women (ages 18–45) who reported on average less than 90 minutes of voluntary moderate- or vigorous- intensity physical activity per week for the past three months. Participants were 338 individuals recruited from the Denver-metro area and the CU community. Individuals were excluded if they smoked cigarettes, were on a restricted diet, taking psychotropic medications, receiving treatment for any psychiatric disorder, diabetic, had a history of cardiovascular or respiratory disease, had the flu or illness in the previous month, or were pregnant (if female). All participants were required to have a body mass index (BMI) between 18 and 37.5, be physically capable of engaging in moderate-intensity physical activity, and have a regular menstrual cycle (if female). All participants had to be willing to be randomly chosen for one of the two interventions and give informed consent. A total of 238 individuals completed baseline assessments and were randomly assigned to condition (see Figure 2).

### Procedure

Following baseline sessions, participants met privately with a trained health educator to be randomized (by coin flip). COSTRIDE participants were instructed in basic physical activity information, goal setting, and physical activity tracking using provided logs. Their goal was to increase their moderate-intensity physical activity to at least five days a week for 30 minutes a day. COSTRIDE participants received mailings with individually-tailored messages and information based on their currently salient barriers to and level of motivation for increasing physical activity at 14 time points: weekly during Month 1, biweekly during Months 2 and 3, monthly during Months 4 through 6, and bimonthly during Months 7 through 12. HW participants were provided with printed materials informing them about various topics (e.g., healthy cooking, stress management, quality sleep) and were told their goal was to increase overall health and well-being. They received non-tailored uniform printed mailings at the same 14 time-points as COSTRIDE participants. Participants were compensated up to $300 for completing the study – receiving increments for completion of each phase. Retention rates differed by follow-up occasion, but were overall consistent with similar exercise trials [17]: 80.3% (*n* = 191), 85.3% (*n* = 203), 67.6% (*n* = 161), and 76.1% (*n* = 181), at the 3-, 6-, 9-, and 12-month follow-ups, respectively. Follow-up retention rates reflect individuals who could not be reached or could not be scheduled for an appointment. Note that due to a clerical error, a number of 9-month assessments were skipped by the research team, and thus the lower retention rate at this wave is primarily data that can be considered missing completely at random (MCAR).

#### Main outcome measures

Physical activity was measured in two ways. The *7-Day Physical Activity Recall* (PAR) [31] interview assessed minutes and intensity of physical activity including voluntary aerobic exercise, work-related activity, leisure-time physical activity, and walking over the previous seven days. This measure has demonstrated reliability and validity [32,33], and is sensitive to changes in moderate-intensity physical activity [9,34]. The 7-day PAR was administered at baseline, 6- and 12-month follow-ups. *Frequency of exercise* of at least moderate intensity was assessed with three questions specifically targeting voluntary aerobic activity of moderate or vigorous intensity [35]. Before answering questions, participants were reminded that the definition of aerobic activity in the current context was “any activity that uses large muscle groups, is done for at least 20 minutes each time, and is done at a level that causes your breathing to be heavy and your heart to beat faster. Examples are running, swimming, bicycling, step aerobics, basketball.” Participants then indicated how often they had engaged in aerobic activity in the past three months (1 = never, 7 = often), the average number of days per week they engaged in aerobic exercise in the past three months (0 days to 7 days), and the number of days they engaged in aerobic exercise in the past week (0 days to 7 days). These items were standardized and averaged, α= .75. The 3-month frequency of exercise measure was assessed at baseline and again at 3, 6, 9, and 12 month follow-ups. Thus there were three time points for the PAR, and five time points for the 3-month frequency measure available for analysis.

#### Fitness test

Participants were instructed to eat a meal equal to at least 300 calories comprised of both carbohydrates and protein and to drink at least 17 oz. of water two hours before both exercise sessions. Participants were instructed not to exercise on their own prior to the laboratory session and not to consume alcohol during the 24 hours prior to testing. Cardiorespiratory fitness was assessed by measuring oxygen uptake using a Balke protocol (a graded maximal exercise test) on a motorized treadmill. Consistent with established procedures [36], maximal oxygen capacity (VO_2_ max) was assessed via online computer-assisted open-circuit spirometry using the Medgraphics Cardi02/CP system (St. Paul, MN) during incremental treadmill exercise (Trackmaster 425 treadmill, Newton, KS). VO_2_max was assessed at baseline and 12-month follow-up in order to provide some objective verification of any self-reported changes in physical activity over the course of the trial. Weight and height were measured for calculation of BMI and saliva samples (5 ml) were collected for DNA extraction before the fitness test. DNA was also collected at the 12-month follow-up to conduct preliminary assessments of epigenetic change due to changes in exercise behavior [37].

#### Submaximal exercise session

Approximately one week after the fitness test, participants completed a 30-minute bout of physical activity on the treadmill at 65% of their previously estimated VO_2_ max. Prior to beginning activity, resting heart rate and blood pressure measures were taken and a nurse inserted an intravenous catheter to collect blood samples during the bout. Intensity was maintained by measuring oxygen uptake and expired CO_2_ for two to three minutes at the beginning of exercise and at 10 and 20 minutes during exercise.

### Moderators assessed during fitness test

#### Genetic factors

At the time of the fitness test, DNA was extracted from saliva using previously published procedures [19]. The well-established Taqman assay from Applied Biosystems, utilized in our previous work [19], was used to assay the *BDNF* SNP (rs6265). Because the samples were eventually assayed on an array (Illumina Human 1 M DuoV3 DNA Analysis BeadChip), we were also able to utilize genotype data from the array (rs9941349, rs8044769, rs3751812, rs2253206, 2360969, rs1799971, rs1935881, and rs1379659).

### Moderators assessed during submaximal exercise session

#### Physiological response to exercise

Blood samples were collected to measure blood *lactate concentration* and plasma *epinephrine and norepinephrine levels* immediately before activity began (11.5 ml), and 10 (5.5 ml) and 30 (11.5 ml) minutes into activity. *Tympanic temperature* was measured by taking an average of 2–3 temperature readings at each measurement. Assessments of temperature and *blood pressure* were taken before activity, three times during activity (10 minutes, 20 minutes, and immediately before the end of activity at 30 minutes), and once five minutes post-activity. Heart rate was continuously monitored to assure that participants were within the range identified as moderate physical activity, and exact heart rate was recorded at the same time as blood pressure and temperature.

#### Subjective experience of exercise

*Perceived pain* experienced during exercise was assessed using a single-item 12-point Borg CR10 scale ranging from 0 (no pain at all) to 10 (extremely intense pain) [38]. *The Feeling Scale* (FS) [39], a single-item 11-point measure (-5 = very bad, +5 = very good), corresponds with the valence component of Russell’s circumplex model of affect [40] and has been used as a measure of general affect during exercise [41]. Subjective response during exercise was measured at the same time as temperature, blood pressure and heart rate.

## Results

### Overview of analyses

All continuous measures were first examined for distributional properties. Only epinephrine exhibited a significant departure from normality. The raw values were log transformed, and the log transformed values were used in all subsequent analyses. Since all analyses were planned *a priori*[42], and because of the difficulty in detecting interactions in field studies [43], critical alpha was maintained at .05 for all analyses. We first explored demographics and baseline physical activity to assess the success of random assignment. We then conducted attrition analyses to determine whether differential attrition generally or by condition occurred across the course of the study. Missing data were approached via the use of full information maximum likelihood (FIML) estimation, whereby all possible data points are utilized and missing values are iteratively estimated using the expectation-maximization algorithm [44,45]. Analyses on intervention outcomes were conducted in a random coefficient regression (RCR) [46] framework via SAS Proc Mixed, which takes into account the longitudinal nature of the data and implements FIML estimation of missing data [44] within the analysis. Moderator analyses were also conducted in a RCR framework.

### Demographics

During the baseline assessment, 19 individuals reported more than 90 minutes of voluntary physical activity in the past week according to the PAR assessment. These individuals were not considered “inactive” and were therefore dropped from the analysis. Thus, the sample used here included 219 individuals. Table 1 provides baseline characteristics across the two intervention groups and outcome variables at 12-months. On average, the sample was 28.20 years of age (*sd* = 7.95). The majority were White (67.1%), were predominantly female (80.4%), had an average of 15.83 years of education (*sd* = 2.60), and more than half had a total household income of $50,000 or more (55.2%). Participants reported engaging in 17.58 minutes (*sd* = 26.58) of at least moderate-intensity exercise over the past week, with a majority (59.8%) being completely sedentary and half (50%) fell into the normal weight range on BMI (18.5-24.9). There were no significant differences between the groups on any baseline demographic data.

**Table 1:** Baseline characteristics and 12-month outcomes by intervention group

	**Health and Wellness (*n*** **= 105)**		**COSTRIDE (*n*** **= 113)**	
	**Baseline**	**12-month**	**Baseline**	**12-month**
Demographics				
Gender (% Female)	81.9		78.8	
Ethnicity (% White)	67.6		66.4	
Age	27.38 (7.81)		28.93 (7.88)	
Physical activity				
Self-report activity (standardized)	.05 (.86)	-.22 (.84)	-.07 (.79)	.17 (.89)
	Range: -1.12 – 3.09	Range: -1.68 – 2.14	Range: -1.12 – 1.76	Range: -1.12 – 2.58
Voluntary exercise minutes (7-day PAR)	16.54 (27.49)	68.42 (72.59)	18.07 (25.45)	103.01 (111.27)
	Range: 0-90	Range: 0-300	Range: 0-90	Range: 0-570
Fitness				
VO_2_max (ml/kg/min)	33.23 (7.10)	33.39 (7.57)	34.07 (8.19)	34.92 (8.49)
BMI	25.52 (5.09)	25.54 (4.94)	24.99 (4.51)	25.55 (4.65)
Resting HR	75.16 (11.75)		74.16 (12.21)	
Resting SBP	114.80 (11.71)		113.51 (13.47)	
Resting DBP	68.90 (8.39)		67.91 (9.42)	

*Note.* No significant differences were found between intervention conditions at baseline.

#### Attrition

Attrition analyses were conducted with regard to who was retained versus not retained at the 12-month follow-up. To examine whether there were differential rates of attrition by condition, a series of ANOVAs were conducted to examine the interaction between attrition at 12-month follow-up (retained vs. not retained) and condition (COSTRIDE versus HW) on pretest measures of key demographic and behavioral constructs [47]. Of 13 tests conducted, there were only two significant effects. Participants with *lower* levels of self-reported frequency of exercise at baseline were significantly more likely to be retained at the 12-month follow-up ( *p* = .004), although this did not differ by condition and baseline levels of behavior are included in every analysis of behavior change over the trial. There was also a significant retentionXcondition interaction for gender, such that males were less likely to be retained in the HW condition (*p* = .02). Thus all main intervention analyses on outcomes were repeated controlling for gender.

### Main effects of the intervention on physical activity outcomes

#### PAR minutes

**Figure 3:**
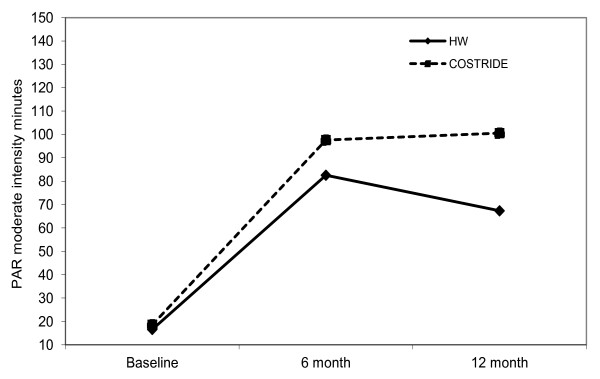
Change in PAR minutes from baseline to 12 months.

In the first analysis, PAR minutes reported at baseline, 6 months, and 12 months served as the dependent measure, and condition (COSTRIDE versus HW) and time were the independent variables. There was a significant timeXcondition interaction on PAR minutes (*est.* = 14.85, *se* = 7.47, *p* = .047). Participants in both conditions increased their moderate intensity physical activity minutes from baseline to 6 months, but those in the COSTRIDE condition appeared better able to maintain those gains from the 6-month to 12-month follow-up (see Figure 3). Simple effects tests confirmed that the COSTRIDE and HW conditions were not different in PAR minutes at the 6 months, but this difference was significant at 12 months (*p* = .02). Outcomes were unchanged after controlling for gender.

#### Frequency of aerobic exercise

**Figure 4:**
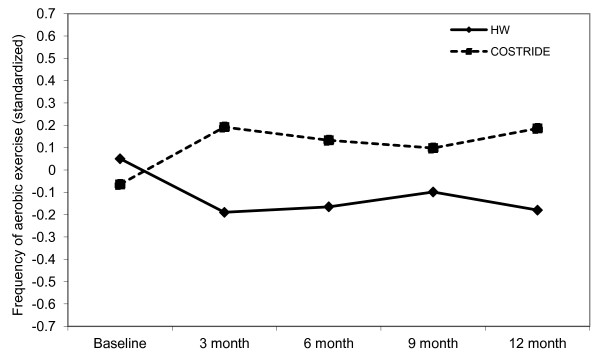
Change in self-report aerobic exercise frequency from baseline to 12 months.

In the second analysis, past 3-month frequency of exercise reported at baseline, 3 months, 6 months, 9 months, and 12 months served as the dependent measure. There was a significant timeXcondition interaction on frequency of exercise (*est.* = .09, *se* = .03, *p* = .006). Participants in the COSTRIDE condition increased their frequency of exercise from baseline to 3 months and then maintained that change over time, while participants in the HW condition actually decreased their frequency of exercise from baseline to 3 months and, similarly, maintained that behavior (see Figure 4). Simple effects tests confirmed that the COSTRIDE and HW conditions were not different in reported frequency of exercise at baseline, nor at the 9 month follow-up. However, the conditions were significantly different at the 3 month (*p* = .002), 6 month (*p* = .02), and 12-month assessments (*p* = .004). The significant intervention effect was unchanged after controlling for gender.

#### VO_2_ max

VO_2_ max at baseline and then at 12 months served as the dependent measure. There was no significant timeXcondition interaction on VO_2_max (*est.* = .30, *se* = .52, *p* = .55). In the HW condition, VO_2_ max went from a mean of 33.23 ml/kg/min (*sd* = 7.10) at baseline to a mean of 33.39 ml/kg/min (*sd* = 7.57) at 12 months. In the COSTRIDE condition, VO_2_ max went from a mean of 34.06 ml/kg/min (*sd* = 8.16) at baseline to a mean of 34.92 ml/kg/min (*sd* = 8.49) at 12 months.

The three outcome measures were most certainly assessing three different, though related, dimensions of physical activity. The correlations between outcomes before and after 12 months of participation in COSTRIDE or HW were high for the self-reported outcomes, but much smaller for VO_2_ max. At both baseline and 12-month follow-up, past 7-day PAR minutes was significantly and positively associated with past 3-month frequency of exercise (*r* = .20, *p* = 004 and *r* = .64, *p* < .001, respectively) and VO_2_ max (*r* = .15, *p* = .03 and *r* = .29, *p* < .001, respectively). Frequency of exercise was not associated with VO_2_ max at baseline (*r* = .08), but was significantly and positively associated at 12 months (*r* = .24, *p* = .002).

### Moderation of intervention effects on physical activity outcomes

For each moderator tested, we estimated a RCR wherein condition, time, the moderator (centered when necessary), and all possible interactions served as the independent variables. The test of primary interest in these analyses is the timeXconditionXmoderator interaction.

#### Genetic factors

**Figure 5:**
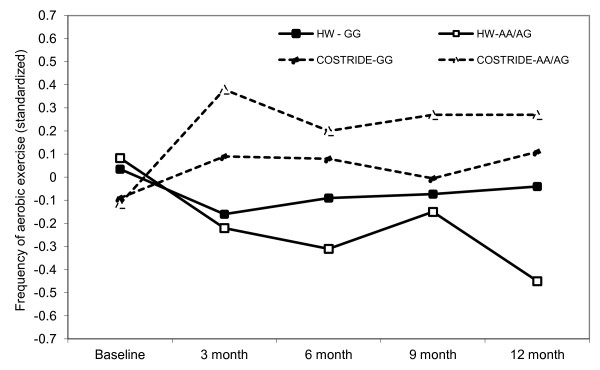
Longitudinal intervention outcomes by BDNF (rs6265): change in frequency of aerobic exercise from baseline to 12 months.

**Figure 6:**
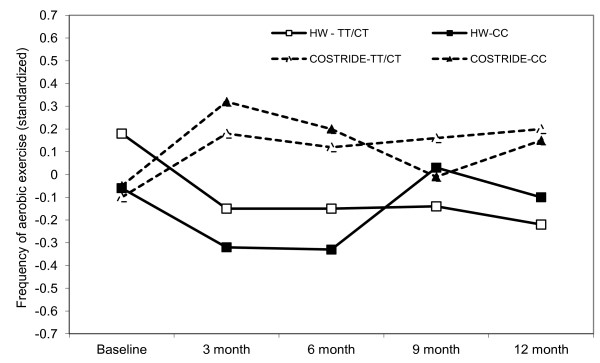
Longitudinal intervention outcomes by FTO (rs8044769): change in frequency of aerobic exercise from baseline to 12 months.

The *BDNF* SNP (rs6265) did not moderate the intervention effects on past 7-day PAR minutes, but it did moderate intervention effects on past 3-month frequency of exercise (*est.* = .14, *se* = .069, *p* < .05). COSTRIDE participants with two copies of the G allele exercised less frequently in the past three months than individuals with at least one copy of the A allele, while individuals with an A allele in the HW condition were the least active (Figure 5). There was no moderation of intervention effects on VO_2_ max by the *BDNF* SNP, however there was a marginal main effect of the SNP on overall levels of VO_2_ max (*est.* = 3.06, *se* = 1.59, *p* = .056). The estimated means indicated that A/A and A/G individuals had higher overall VO_2_ max (*M* = 34.99, *se* = .84) than G/G individuals (*M* = 33.58, *se* = .64). There was no moderation of intervention effects on 7-day PAR minutes by a SNP in the *FTO* gene (rs8044769). However, there was evidence for the moderation of intervention effects on past 3-month frequency of exercise by this FTO SNP (*est.* = -.15, *se* = .076, *p* = .05). COSTRIDE participants with two copies of the C allele exercised more frequently, while individuals with at least one copy of the T allele exercised less frequently (Figure 6). There was no moderation of intervention effects on VO_2_ max by this FTO SNP. Additionally, there was no moderation of intervention effects on PAR minutes, voluntary aerobic exercise, or VO_2_ max by the other SNPs examined.

#### Physiological responses to exercise

In order to explore the extent to which physiological responses to exercise moderated intervention outcomes, it was necessary to compute slopes that characterized the response during exercise for each of our physiological moderators. We utilized a RCR framework in SAS Version 9.2 to model linear changes in the response over the course of the submaximal exercise challenge. These analyses produced individual within-subject regression slopes that characterized each individual’s change in, for example, heart rate over the course of the bout of exercise. These slopes then served as an additional predictor in the overall RCR models assessing intervention effects. There was no significant moderation of intervention effects on past 7-day PAR minutes, past 3-month aerobic exercise, or VO_2_max by lactate, temperature, systolic blood pressure, norepinephrine or heart rate response to a bout of moderate intensity exercise.

#### Subjective experience of exercise

Next, the extent to which the subjective experience of moderate intensity exercise moderated intervention outcomes was tested. The same strategy of computing within-subjects regression slopes for each of our measures of subjective experience was utilized. There was no significant moderation of intervention effects on past 7-day PAR minutes, past 3-month aerobic exercise, or VO_2_max by perceived pain or affective response to a bout of moderate intensity exercise.

## Discussion

The STRIDE intervention was successfully replicated in a geographically different sample of sedentary individuals. Although all intervention participants increased minutes of physical activity in the past week from baseline to the six-month follow-up, COSTRIDE participants were able to maintain a higher level of physical activity through 12 months. Additionally, more global assessments of physical activity over the past three months indicated that COSTRIDE participants were generally better at maintaining higher levels of voluntary aerobic exercise throughout the course of the study. Although there were promising self-reported behavioral differences, there was no effect on objective fitness (VO_2_ max), and little average change in VO_2_ max. This suggests that while our participants had been increasing their minutes of physical activity and the frequency with which they engaged in these activities, they may not have been working at an intensity level high enough to result in increased VO_2_ max. Of course, given the reliance on self-report, it could also be that the frequency and volume of activity reported by participants was overestimated and that they were not engaging in sufficient amounts of overall participation to yield meaningful changes in cardiovascular fitness. The exercise physiology literature is clear that it is higher total exercise volume (i.e., higher *intensity* exercise for longer durations) rather than simply a longer time spent active, that is associated with the greatest effects on cardiovascular fitness [48-51]. Despite less than optimal effects on cardiovascular fitness, physical activity among sedentary individuals did increase to a level that has a myriad of other health benefits (e.g., cancer prevention, psychological benefits, metabolic syndrome prevention).

There was some evidence for the moderation of intervention effects by genetic factors, in that we showed effects of two SNPs that have been previously associated with response to exercise [19,21]. The findings extend prior work by demonstrating stronger intervention response among individuals with a genotype associated with more positive affective response to acute exercise. Specifically, in prior work *BDNF* A/A and A/G individuals perceived less exertion during moderate intensity exercise and experienced more positive affect in response to exercise than G/G individuals [19], and the A/A or A/G individuals were more likely to exercise in the current study in the intervention condition, but less likely to exercise in the HW condition. With regard to the *FTO* SNP, C/C individuals in this same sample experienced more positive affect in response to exercise than C/T and T/T individuals at baseline [21]. Our findings regarding more frequent exercise behavior among C/C individuals in response to the intervention are consistent with this relationship. Interestingly, it appears that the effects were most pronounced for the *FTO* SNP in the first six months, and were less apparent in the second six months, suggesting that this particular SNP may be more strongly associated with exercise initiation rather than maintenance. Although the effects demonstrated here are arguably rather small, they do support recent ideas about the potential for genetic moderation of intervention effects in the behavioral domain similar to what has been found in the pharmacological domain [52,53].

Despite relationships between physiological responses, subjective responses, and exercise motivation and behavior shown in other work [22-24], there was no evidence that physiological or subjective responses to an acute bout of moderate intensity exercise moderated effects of the intervention. There are at least two possibilities to account for why these moderating outcomes were not found. The first possibility is that the intervention itself is essentially an “ongoing dose” of intervention over the full 12 months of the behavioral follow-up. This situation may simply dampen individual differences in behavior that might naturally arise when constant external reinforcement is not provided. Whereas an individual who is intrinsically motivated by a positive affective response to exercise will continue to be physically active, an individual who perceives less positive affective response to exercise might simply stop exercising in the absence of encouragement from external sources. Such a condition did not exist in the current study. The second possibility is that these physiological and subjective responses to exercise simply do not moderate response to an exercise intervention.

The overall results of our study could potentially be viewed as “underwhelming” in terms of the lack of significant moderation effects, and we are certainly not the first to show somewhat disappointing findings with regard to the strength of moderators of exercise intervention efficacy. Importantly, however, we view our approach as methodologically stronger than existing work in that our moderators were drawn from a theoretically plausible and empirically supported transdisciplinary model of exercise behavior. In contrast, prior investigations have taken an atheoretical approach, and focused largely on demographic (e.g., gender), motivational (e.g., self-efficacy), or personality (e.g., depression) factors that were measured prior to engaging in an intervention. Here, the focus was on physiological and subjective responses experienced *during* an acute bout of moderate-intensity physical activity and genetic factors that might moderate intervention efficacy. To our knowledge, this is the first investigation into theoretically grounded underlying determinants of physical activity as moderators of the initiation and maintenance of exercise in an RCT. Yet, despite our methodological rigor, the results did not reveal any stronger pattern of moderation than prior studies.

Are we to conclude, then, that focusing on moderators of physical activity intervention efficacy is unimportant? We think this conclusion might yet be premature. At this point, all of the empirical tests for moderation of intervention effects on physical activity (including the current RCT) have compared a physical activity intervention to either a no-treatment control, an information-only control or an attention-placebo control [15-18]. It is possible that the individual differences that influence response to physical activity intervention are not so much related to whether an individual receives an intervention or not, but rather *what kind* of physical activity intervention one receives. The intervention utilized in this study involves ongoing tailored feedback which has been shown via meta-analysis to be a successful technique [12]. Other successful interventions involve self-monitoring (observing and processing information concerning internal and external states) as an effective strategy for maintaining physical activity behavior [54,55]. It is possible that any of these interventions are strongly superior to information only or no-treatment controls, such that they overwhelm individual differences moderating their efficacy. Studies that compare *different exercise intervention strategies* to one another may afford a context in which moderators may show stronger effects. Perhaps an individual with positive affective response to exercise responds better to immediate engagement in levels of intensity and duration that maximize the positive affect response, while those who experience greater temperature increase and pain may respond better to gradual introductions to exercise and a more extensive suite of external reinforcement contingencies. Indeed, in the current study, there is preliminary evidence that moderators of exercise *initiation* may differ from moderators of exercise *maintenance*. Finally, it is interesting to note that while two of the genetic moderators were significant, none of the moderators classified as “responses to physical activity” were moderators of intervention effects. We speculate that perhaps the reason for this is that these reactions were measured during a single bout of physical activity and might display significant variability over time depending on any number of factors. Meanwhile, genotype does not vary, perhaps making it a more reliable potential moderator of intervention effects over time. Clearly additional research is necessary to understand the degree to which immediate responses to physical activity are consistent over time.

The current study has many strengths including the use of an exercise intervention with well-documented success, the design, the recruitment of sedentary individuals, a theory-based conceptual framework as the basis for our selection of potential moderators, the inclusion of moderators across the transdisciplinary spectrum in the same study, the frequency of follow-up assessments, and the length of follow-up. However, this study is limited by a relatively small sample for testing moderated effects, the nature of the sample (mostly women), and the lack of objective verification of self-reported exercise. We originally included accelerometry on a subsample of participants as our budget constraints allowed. We found the data was essentially equivalent to PAR data [56]. Due to higher than expected costs, subject burden involved in returning the units, the ability to only sample a subset of participants, and the lack of added gain in measurement, accelerometer use was discontinued in the early stages of the project. We are also limited by our exclusive focus on intra-individual factors as moderators. Clearly, the environment, sociocultural context, and neighborhood safety, are crucially important in accounting for variability in exercise behavior [57]. Finally, we conducted a number of statistical tests, raising the potential concern of alpha inflation. Thus, it is important—particularly in the context of our moderated effects—that our results be interpreted with caution until they are replicated.

There is increasing interest in the extent to which genetic information can be harnessed to promote engagement in health behavior in general [53] and in exercise behavior more specifically [58,59]. Genetic factors account for substantial variability in both engagement in leisure time physical activity [60] as well as physiological responses to exercise training [61]. The current work provides initial evidence that genetic factors may underlie *behavioral* responses to physical activity interventions. But can understanding an individual’s genetic profile help researchers and health professionals to understand how they will respond to a behavioral intervention to increase exercise? At least one study has demonstrated a genetic moderator of responses to a behavioral intervention to reduce alcohol abuse [62], but the science of intervention targeting and tailoring based on genotype is in its infancy. While individual genes accounting for small amounts of variation in intervention response provide proof of concept, it is ultimately much more likely that a combination of hundreds or even thousands of genetic markers will provide both greater statistical power for detecting moderation and the possibility of increased clinical utility of genomic information for the targeting and tailoring of exercise intervention.

## Competing interests

The authors declare they have no competing interest.

## Authors’ contributions

ADB is the principal investigator, designed the study from which these data are derived, and directed study implementation. BM and JTC contributed to the verification of the successful implementation of the STRIDE intervention. REM, AEH, and ADB contributed to data analysis, planning of the research questions, and interpreting results. REM, AEH, JTC, and ADB wrote portions of text for the manuscript. KEH oversaw genetic analysis. All authors have read and approved the final manuscript.
